# Changes in urinary metabolomic profile during relapsing renal vasculitis

**DOI:** 10.1038/srep38074

**Published:** 2016-12-01

**Authors:** Bahjat Al-Ani, Martin Fitzpatrick, Hamad Al-Nuaimi, Alice M. Coughlan, Fionnuala B. Hickey, Charles D. Pusey, Caroline Savage, Christopher M. Benton, Eóin C. O’Brien, Declan O’Toole, Ken H. Mok, Stephen P. Young, Mark A. Little

**Affiliations:** 1 Renal Immunobiology Group, School of Infection, Immunology and Inflammation, University of Birmingham, UK; 2 Rheumatology Research Group, Centre for Translational Inflammation Research, College of Medical and Dental Sciences, University of Birmingham, UK; 3 Department of Clinical Medicine, Trinity College Dublin, Ireland; 4 Renal Section, Imperial College London, London, UK; 5 Agilent Technologies Ltd, UK; 6 School of Biochemistry and Immunology, Trinity Biomedical Sciences Institute (TBSI), Trinity College Dublin, Ireland; 7 Trinity Health Kidney Centre, Trinity Centre for Health Sciences, Tallaght Hospital, Dublin 24. Ireland

## Abstract

Current biomarkers of renal disease in systemic vasculitis lack predictive value and are insensitive to early damage. To identify novel biomarkers of renal vasculitis flare, we analysed the longitudinal urinary metabolomic profile of a rat model of anti-neutrophil cytoplasmic antibody (ANCA) vasculitis. Wistar-Kyoto (WKY) rats were immunised with human myeloperoxidase (MPO). Urine was obtained at regular intervals for 181 days, after which relapse was induced by re-challenge with MPO. Urinary metabolites were assessed in an unbiased fashion using nuclear magnetic resonance (NMR) spectroscopy, and analysed using partial least squares discriminant analysis (PLS-DA) and partial least squares regression (PLS-R). At 56 days post-immunisation, we found that rats with vasculitis had a significantly different urinary metabolite profile than control animals; the observed PLS-DA clusters dissipated between 56 and 181 days, and re-emerged with relapse. The metabolites most altered in rats with active or relapsing vasculitis were trimethylamine N-oxide (TMAO), citrate and 2-oxoglutarate. Myo-inositol was also moderately predictive. The key urine metabolites identified in rats were confirmed in a large cohort of patients using liquid chromatography–mass spectrometry (LC-MS). Hypocitraturia and elevated urinary myo-inositol remained associated with active disease, with the urine myo-inositol:citrate ratio being tightly correlated with active renal vasculitis.

Microscopic polyangiitis (MPA) is a relapsing autoimmune condition characterised by necrotising angiitis usually involving the glomerulus and frequently resulting in rapidly progressive kidney failure. It is associated with the presence of anti-neutrophil cytoplasm antibodies (ANCA) primarily directed against myeloperoxidase (MPO)^1^,^2^. The adverse events of therapy may be as big a clinical problem as the disease itself 3. Indeed, over half of those who die within the first year of diagnosis do so as a result of infection, presumably due to over-immunosuppression. One of the main challenges impinging upon this balancing act between over and under-treatment is accurate assessment of disease activity. This is particularly true during follow up when one must always make a judgement as to whether disease is active or quiescent. Current biomarkers assisting in this decision include urinalysis to detect haematuria and proteinuria, C-reactive protein (CRP) to detect active systemic inflammation, evidence of end-organ dysfunction (such as rising serum creatinine), and anti-MPO antibody titre. These are very poor biomarkers, being either insensitive (once the creatinine level rises out of the normal range, approximately 30% of kidney function has been lost), non-specific (haematuria may derive from other conditions such as urinary tract infection or cyclophosphamide-induced cystitis), or of poor predictive value (anti-MPO antibody levels correlate very poorly with disease activity^4^). Therefore, an important unmet need in this relapsing disease is a non-invasive test to accurately determine the current level of disease activity.

We used a urinary metabolomic approach to identify potentially useful clinical biomarkers of active renal vasculitis. Metabolomics has been successfully employed for the identification of biomarkers in a number of diseases and models, including urinary tract infection, ulcerative colitis, rheumatoid arthritis, multiple sclerosis^5^,^6^,^7^, and both human renal disease and rat models of tubular disease and acute kidney injury^8^. The experimental autoimmune vasculitis (EAV) model in the Wistar-Kyoto (WKY) rat was used to define serial changes in the urine metabolome as the disease evolves, and to test the ability of this approach to distinguish between animals in remission and relapse. We then investigated whether similar urinary metabolites were informative in patients with vasculitis.

## Results

### Evolution of renal vasculitis and scarring in rats with EAV

**Figure 1:**
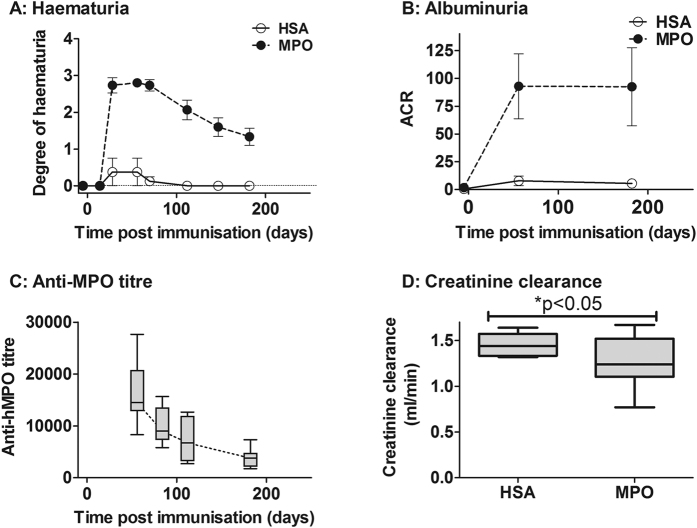
Change in urine parameters and anti-MPO titre over time . WKY rats were immunised with hMPO or HSA, and followed for 181 days. **(A)** Haematuria peaked at 56 days post immunisation and declined steadily thereafter. **(B)** Albuminuria, as assessed by albumin:creatinine ratio (ACR), also peaked at day 56, but remained elevated until day 181. Data are presented as mean +/− s.e.m. **(C)** Anti-MPO titres declined progressively over time and rats with EAV exhibited reduced excretory renal function, as estimated by measurement of (**D**) creatinine clearance. Data are presented as median and IQR.

WKY rats were immunised with human (h) MPO (EAV; n = 12) or human serum albumin (HSA) (Control; n = 7), and assessed at regular intervals for a period of 181 days. Urinary markers of active renal vasculitis (haematuria and albuminuria – reported as the albumin to creatinine ratio [ACR]) along with anti-MPO titre peaked at 56 days post immunisation (Fig. 1). Haematuria and anti-MPO titre declined progressively thereafter while albuminuria remained elevated to a similar level throughout the period of observation. Excretory renal function, as quantified by creatinine clearance, was reduced in EAV animals at 181 days (Fig. 1D). To assess whether EAV rats developed renal scarring (as is observed in patients with MPO-ANCA vasculitis) we stained renal tissue with periodic acid Schiff (PAS)-silver and picrosirius red. At 30 weeks post-immunisation (day 210), EAV animals exhibited increased renal scarring when compared to the same animal at week 8 (day 56) (Supplemental Fig. S1) and to control animals (data not shown). Most of the scarring was in a periglomerular distribution.

### Active vasculitis is associated with a distinct urinary metabolite profile

**Figure 2:**
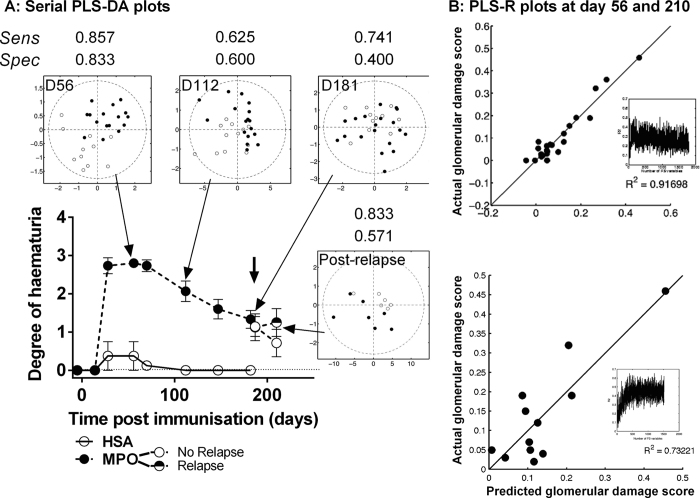
Urinary metabolite profile of rats with EAV at the point of peak disease, during remission and following induction of relapse. (**A**) PLS-DA on 2 Latent variables at several time points during the evolution of EAV (day 56, day 112, day 181 (pre relapse) and day 210 (post relapse)) discriminating between (●) MPO (n = 16) and (○) HSA (n = 11) groups, and between MPO re-stimulation (◓, n = 6) or control (saline) re-stimulation (○, n = 7) of EAV animals. MPO and HSA separate on 2 LVs following stimulation; this separation is largely lost by day 112 and 181, with re-emergence following induction of relapse. Haematuria data are presented as mean +/− s.e.m. (**B**) PLS-R of binned 1D NMR spectra of rat urine against histological glomerular damage score identifies key urine metabolite markers predictive of damage at day 56 (upper plot) and day 210 (lower plot). The correlation plot shows predicted vs. observed damage scores using N bins.

To determine if urinary metabolomic analysis could distinguish between MPO and HSA immunisation groups, serial urines were analysed by nuclear magnetic resonance (NMR). Partial least squares-discriminant analysis (PLS-DA) of the resulting spectra demonstrated separation of HSA and MPO immunised animals at the point of peak disease (day 56) (Fig. 2A). PLS-DA weightings showed that the key peaks associated with separation at this time point were trimethylamine N-oxide (TMAO), 2-oxoglutarate and citrate, with contribution also from di-methyl glycine (DMG), succinate, homoserine, betaine and myo-inositol (Table 1). We next sought to relate the metabolic data obtained at day 56 to the degree of glomerular injury in animals sacrificed 154 days later. We performed a partial least squares regression (PLS-R) of urinary NMR spectral bins against the histologically determined glomerular damage scores. Despite the long interval between metabolite assay and histological quantification the resulting model gave a strong and significant fit (P < 0.05; R^2^ = 0.9313) (Fig. 2B). Bins were ranked according to their relative contribution to regression fit, identifying TMAO, 2-oxoglutarate, and succinate as key predictors of glomerular injury (Table 1; Supplemental Fig. S2).

### The urine metabolite profile provides an improvement to existing markers

We next sought to confirm these putative urinary markers of disease activity against existing standards – dipstick haematuria and ACR. At day 56 ACR and haematuria were significant discriminators between MPO and HSA immunised animals (Supplemental Table S1). Metabolites DMG and TMAO remained significantly predictive in this analysis. All 4 markers were taken forward for logistic regression model building. Haematuria and ACR resulted in a model with 90.5% accuracy (Table 2). Adding metabolite markers DMG and TMAO to the model raised the predictive accuracy to 95.2%.

We also assessed whether the metabolic changes observed at day 56 persisted, by examining urine samples taken at days 112 and 181 post-immunisation (Fig. 2A). Analysis at day 112 showed a reduced PLS-DA model fit and this was further reduced by day 181. While 74% of MPO-immunised animals were correctly classified at day 181, 60% of HSA-immunised animals were misclassified using this model.

### Induction of relapse in EAV animals

**Figure 3:**
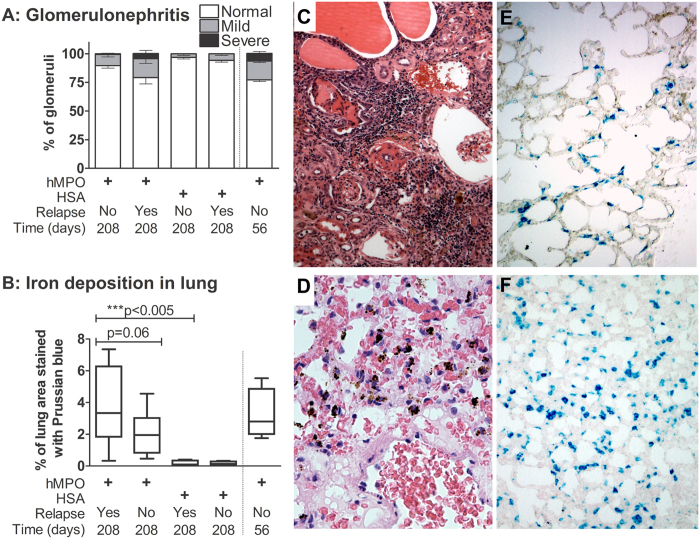
Effect of relapse induction. WKY rats were immunised with hMPO or HSA and sacrificed at day 56 or day 210, the latter following treatment with either LPS + MPO (Relapse) or saline (Control) at day 181. (**A**) The degree of glomerulonephritis was scored blindly at 56 days and 210 days. **(B)** Lung haemorrhage was assessed by staining lung sections with Perl’s stain, which shows iron deposition as blue, and quantifying the degree of blue staining with image analysis. Data are presented as median and IQR. **(C)** Representative image of a renal section from a severely affected rat relapsed with MPO and LPS. There is a marked interstitial infiltrate, focal necrotising crescentic glomerulonephritis and tubular dilatation (H&E, x100). **(D)** Representative image depicting brown-staining haemosiderin-laden alveolar macrophages in a lung section from a relapsed animal (H&E, x400). **(E**,**F**) Representative images from Perl’s stained lung sections from **(E)** a control and **(F**) a relapsed animal (x10).

At 26 weeks post initial immunisation (day 181), EAV rats were re-treated with soluble hMPO plus LPS (EAV relapse; n = 7) or saline (EAV no relapse; n = 7). In the relapse animals, the decline in anti-MPO titre and haematuria was halted, but there was no significant difference in absolute levels compared to controls (data not shown). The degree of glomerulonephritis was similar between EAV relapse animals (sacrificed on day 210) and animals sacrificed at day 56, while there was virtually no severe glomerular injury or crescents in the non-relapsed animals (Fig. 3A,C). We quantified alveolar haemorrhage by measuring the amount of respective lung section staining blue with Perl’s stain, which detects intra-vital deposited iron (Fig. 3B,D–F). Blue staining cells (presumed to be haemosiderin-laden macrophages) were present in the walls of the alveoli (Fig. 3D). EAV relapse animals displayed significantly higher levels of blue staining than HSA-immunised animals (Fig. 3B, E-F). There was a non-significant trend towards greater staining in EAV relapse animals compared to EAV non-relapsed animals (Fig. 3B).

### Induction of relapse is associated with a partial re-emergence of the vasculitic urine metabolite profile

Having determined the phenotype of relapse using established histological markers, we sought to assess if this was mirrored by the urinary metabolite profile 29 days post induction of relapse (day 210). Analysis of urine samples indicated a return to separation by PLS-DA model (Fig. 2A). The metabolic markers were broadly similar to those observed at day 56, with citrate and 2-oxoglutarate being the strongest discriminators between the two groups (Table 1). However, TMAO, while present, was now ranked considerably lower. As before, we used PLS-R analysis to relate metabolites to histological damage using histological glomerular damage score on tissue obtained one day after the urine collection. This confirmed the presence of the same metabolites (Fig. 2B, Supplemental Fig. 2) with TMAO the strongest correlate of glomerular injury by PLS-R. Dimethylamine, citrate, maltose and 2-oxoglutarate were also discriminatory with less power.

### Metabolite markers improve disease activity prediction over haematuria and proteinuria

To assess whether addition of urine metabolites to existing markers (haematuria and ACR) improved the ability to detect relapse, we tested for differences between MPO and control relapsed groups and went on to build a binary logistic model as performed at day 56 (Supplemental Table S2; Table 3). Of note, haematuria and ACR were not significantly different between relapse and control groups at this disease point. Of the proposed urine metabolite markers only 2-oxoglutarate was significantly different. Haematuria, ACR and 2-oxoglutarate were taken forward into a binary logistic model. Here haematuria and ACR performed poorly, with a prediction accuracy of 58.3% (p = 0.665). Addition of 2-oxoglutarate improved predictive performance and accuracy; however, the strongest model used 2-oxoglutarate alone, reaching 83.3% accuracy for prediction (p < 0.05).

### Metabolite markers identified in EAV are detectable in patient urine by LC-MS

**Figure 4:**
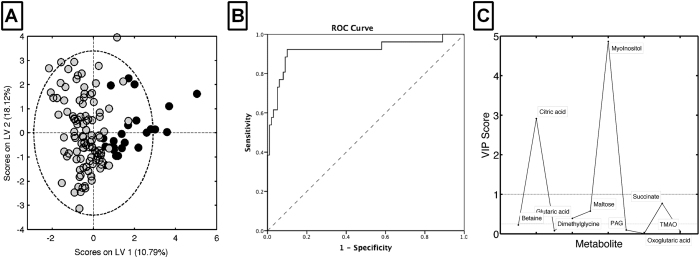
Urinary metabolite profile of patients with active and remission vasculitis. (**A**) PLS-DA of binned LS-MS spectra of human urine samples from patients with active renal vasculitis (●, n = 28) against cases in remission (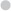, n = 104). PLS-DA weightings identify key metabolites associated with active renal vasculitis. (**B**) Receiver operator characteristic curve showing the prediction accuracy of the binary logistic model based on citric acid and myo-Inositol with area under the curve (AUC) = 0.922 (95% CI 0.849–0.970, p = 0.001). (**C**) Variable importance in the project (VIP) scores summarising the predictive value of each metabolite in identifying active renal vasculitis. Values above 1 indicate a useful marker.

We then sought to apply the unbiased metabolomic findings of the animal study to assess whether a targeted metabolomic analysis could identify active renal vasculitis in patients (Supplemental Table S3). Nine of the metabolites identified from the EAV study were selected and targeted for LC-MS analysis of patient urine, all of which were successfully identified and quantified. Four of the targeted metabolites were found by PLS-DA and ANOVA to be significantly different between patients with active renal vasculitis and those in remission — myo-inositol, citric acid, maltose and succinate (Fig. 4A; Table 4). These metabolites, all of which had a variable importance in the project (VIP) score> 0.25 (Fig. 4B), and existing clinical markers were taken forward for subsequent analysis.

### The myo-inositol:citric acid ratio is highly predictive of active renal vasculitis

**Figure 5:**
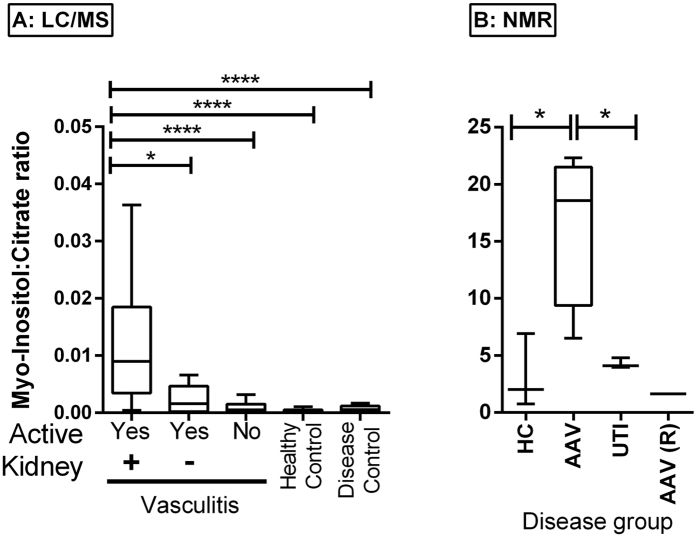
Utility of the urine myoinositol:citrate ratio in identifying active vasculitis. (**A**) Myo-inositol:citrate ratio as determined by LC-MS according to diagnostic groupings. (**B**) Reverse confirmation of urine myo-inositol:citrate ratio using NMR, with inclusion of patients with urine infection (UTI), N = 3–4 per group. A value from a patient with remission vasculitis is shown for comparison. Bars depict median and 90^th^ centile. AAV (R) = AAV in remission. ****p < 0.001, *p < 0.05.

Urine metabolomic markers were added to a binary logistic regression model to test the ability to predict active renal vasculitis among patients with known vasculitis. The resulting model consisted of two metabolites – citric acid and myo-inositol (Table 5). Additional models were built for comparison of existing markers – CRP, estimated glomerular filtration rate (eGFR) and protein:creatinine ratio (PCR). Existing marker prediction accuracy for identification of active renal vasculitis was 89.6%, metabolites alone was 91.7%, and a model combining existing markers with metabolites was 95.8%. The accuracy of the combined model was confirmed using a bootstrapping analysis (Supplemental Table S4). Stratification by ANCA specificity (proteinase 3 (PR3) v MPO) did not affect predictive capacity (data not shown). A Receiver Operator Characteristic (ROC) curve was constructed to assess the performance of the metabolite model in identifying active renal vasculitis (Fig. 4C). As citric acid was decreased in the urine of patients with active renal vasculitis, while myo-inositol was elevated, we investigated the association between the myo-inositol:citric acid ratio and disease state (Fig. 5A). This ratio was markedly elevated in active renal vasculitis when compared to patients in remission, healthy controls, and disease controls; a smaller signal was also observed in patients with active extra-renal vasculitis. As patients received increasing amounts of therapy the myo-inositol:citrate ratio appeared to decline (Supplemental Fig. 3) and patients classified blindly as in remission but receiving induction therapy with cyclophosphamide or rituximab had a slightly elevated value. This probably reflects persistent sub-clinical disease in the context of ongoing induction therapy. To confirm the findings further against urine from patients with kidney infection, we used NMR to compare to the myoinositol:citrate ratio in active renal vasculitis (Fig. 5B), which confirmed the specificity of the LC-MS findings.

## Discussion

Although the ANCA test is an excellent biomarker in diagnosing systemic vasculitis, it performs poorly when determining whether the vasculitic process is active or not. The unmet need in this field is a test that determines when glomerular inflammation has stopped (allowing discontinuation of induction therapy), and that provides a sensitive means of detecting re-emergence of glomerulonephritis in the setting of relapsing disease. We modelled the chronic relapsing phenotype of MPO-ANCA vasculitis in an effort to identify urinary metabolites that reflect active inflammation more accurately than measurement of urinary blood and protein. WKY rats immunised with hMPO developed pauci-immune glomerulonephritis with peak effect at day 56, after which the immune response and glomerular damage declined progressively over the subsequent 17 weeks. Although there was no significant increase in haematuria after re-challenge with MPO plus LPS, when the kidneys were analysed 154 days later, the glomerular injury resembled that at day 56, whereas non-relapsed animals at the same time point had very mild glomerular disease. We went on to analyse the urinary metabolomic profile at various time points during the disease course and found that the marked separation between MPO and HSA immunised animals at day 56 had largely disappeared by day 181 (immediately prior to re-challenge), and re-emerged after re-challenge with the antigen and LPS. Addition of metabolite markers to measurement of haematuria and proteinuria increased the correct classification of animals into relapsed versus non-relapsed. We confirmed our findings in patients with vasculitis by using LC-MS to analyse a targeted selection of the metabolites that we had identified in the rat. Although there were some differences between the rat and human data, this targeted approach allowed us to identify myo-inositol and citrate, and more specifically the ratio between these two metabolites, as key biomarkers associated with active renal vasculitis.

Our analysis showed highest rank bins in the acute response were associated with TMAO, a previously identified marker of renal medullary injury^9^,^10^, 2-oxoglutarate, citrate, DMG and succinate. TMAO is a product of the oxidation of trimethylamine derived from choline metabolism. Its role as a marker of renal medullary injury may be an indicator of the tubulointerstitial nephritis that usually co-exists with the glomerular injury in this rat model.

Citrate, succinate and 2-oxoglutrate are major citric acid cycle metabolites and, although we cannot prove this with our data, a possible indicator of high energy demands, either resulting from renal repair or infiltrating immune cells. Of particular relevance in this respect is the recent discovery of aerobic glycolysis (“Warburg effect”) in activated immune cells, such as M1 macrophages and Th17 lymphocytes^11^,^12^. Under inflammatory conditions, the citric acid cycle appears to be arrested in these cells, with accumulation of succinate and other intermediates. The citric acid cycle signal evident in rats with active disease, particularly the consistent finding of 2-oxoglutarate, is consistent with the concept of aerobic glycolysis in infiltrating glomerular leukocytes, but would require direct assay of these metabolites in renal tissue to delineate further.

Although it was important to demonstrate a distinct metabolic profile in acute disease, the primary focus of this work was on changes in the urine metabolome as the pathology dissipated and relapsed. To model this we defined remission at 181 days post-immunisation with MPO. By this time the EAV and control groups were virtually inseparable by PLS-DA of urinary metabolites. At this point the EAV rats were re-stimulated with MPO or mock-stimulated with saline and urine samples were compared 29 days later. Re-stimulation with MPO was associated with a urine metabolic profile similar to that at day 56. Citrate and 2-oxoglutarate were the strongest differentiators of relapse, with TMAO being no longer predictive. However, despite this finding, PLS-R against glomerular damage score again showed TMAO to be the strongest predictor of histological injury, suggesting that the urinary TMAO signal may reflect the totality of renal parenchymal injury rather than recurrence of glomerular inflammation in the context of experimental vasculitis flare. We next sought to rate theses metabolites against existing biomarkers of renal damage (ACR and haematuria) using binary logistic models. While haematuria and ACR proved strong predictors of active disease at day 56, they proved a poor marker of relapsing disease. In relapsing disease, we identified a single predictive metabolite, 2-oxoglutarate, which successfully identified relapsed animals.

Having identified a set of metabolites offering diagnostic potential in the EAV model, we sought to confirm this in human vasculitis. A small number of metabolites were analysed based on the unbiased rat study allowing us to use a large number of urine samples comprising several different experimental groups and to use a different technique, LC-MS, which is more sensitive than NMR. Although, there were common metabolites between rat and human samples, there were notable differences, such as the absence of TMAO, DMG and 2-oxoglutarate in the human analyses. However, the reciprocal association between urinary citrate/myo-inositol and active vasculitic glomerulonephritis transferred to the human setting, performing better than existing markers CRP, eGFR and PCR. We did not obtain sufficient data on haematuria in the included patients to include this in the analysis. The combination of metabolites, CRP, eGFR, and PCR correctly allocated cases in over 95% of cases and the ratio of citrate:myo-inositol separated active renal vasculitis from other groups with a high degree of precision. A small signal was observed with the metabolite ratio in patients with active extra-renal vasculitis despite having no evidence of glomerulonephritis, suggesting that the urinary value also partially reflects filtered citrate and myo-inositol.

To conclude, we have demonstrated the predictive ability of a pair of urine metabolite markers, citrate and myo-inositol, that may have clinical value in the diagnosis of active renal vasculitis.

## Methods

### Ethics Statement

All animal experiments were performed according to UK home office regulations under project license number 400/3228, and with full approval from the University of Birmingham Ethical Review Committee. Human urine samples were obtained from the Irish Rare Kidney Disease (RKD) Biobank, and written informed consent was obtained from all individuals. The RKD Biobank has full approval from the Trinity College Dublin Institutional Review Board and Ethics Committee, and approval from each of the individual hospitals from which samples were obtained (St. James’ hospital, Tallaght hospital and Beaumont hospital). All experiments were carried out in accordance with the approved guidelines.

### Induction of EAV and relapse

Female WKY rats (Charles River, UK) (100–130 g) were immunised on day 0 with a total of 3.2 mg/kg hMPO (a kind gift from Biovitrum, Stockholm, Sweden) or human serum albumin (HSA) in an equal volume of complete Freund’s adjuvant (CFA, Sigma-Aldrich, UK) at 2 intramuscular and 2 subcutaneous sites. All animals received 1μg of pertussis toxin i.p. (Sigma-Aldrich, UK) on days -5 and 0, and 1500 EU/g lipopolysacharide (LPS) on days 7, 28, and 35. Booster doses (100 μg/kg) of soluble MPO or HSA were administered i.p. on days 21 and 35. 181 days post-immunisation we induced relapse with LPS (1500 EU/g) plus 100 μg/kg MPO or HSA in controls. Non-relapsed animals received saline. Animals were culled 29 days post-relapse.

### Assessment of disease phenotype

We used haematoxylin and eosin (H&E) and periodic acid Schiff (PAS)-stained sections to quantify glomerulonephritis and tubulitis. 50 glomeruli per section were scored blind as normal, abnormal or severely diseased. Picrosirius red staining of tissue sections was used to quantify renal scarring. Embedded kidney sections were dewaxed in xylene and rehydrated through a series of graded alcohols to water. Slides were incubated overnight with 0.1% Sirius red (Sigma-Aldrich, UK), quickly dipped in 0.01 M hydrochloric acid and dehydrated with serial ethanol concentrations without water. The overall degree of staining was quantified in Image J after excluding blood vessels. Kidney fibrosis was visualised by PAS stain with the addition of methenamine silver solution.

We used Perl’s stain to identify haemosiderin laden macrophages in the lung^13^ allowing differentiation of pre and post-mortem bleeding. Embedded lung sections were dewaxed and hydrated to distilled water. Slides were placed for 20 minutes in freshly prepared working solution (equal volume of 2% hydrochloric acid and 2% potassium ferrocyanide) and washed in tap water. Sections were dehydrated with alcohol and cleared with xylene. An automated staining machine (Dako Artisan) was used to perform Perl’s stain. Images were captured and analysed in Aequitas IA image analysis software (Dynamic Data Links, Cambridge, UK).

### Quantification of urinary metabolites by NMR

Using metabolic cages, rat urine samples were collected for 24 hours in containers with 200 mM boric acid added to prevent bacterial growth. The presence of blood and leukocytes was assessed by dipstick, with a semi-quantitative score ranging from 0–4. Urine samples were sterilised by filtration through a 0.22 μm pore. We measured urinary albumin excretion rate by Nephrat ELISA (Exocell, Philadelphia, USA), and creatinine was measured by the modified Jaffe reaction in an autoanalyser. Urinary albumin was reported as the albumin to creatinine ratio (ACR). Samples for NMR were buffered with phosphate buffer (100 mM), containing 10% D_2_O and 0.5 mM TMSP and the pH adjusted (twice over 30 mins) to pH 7.0. Samples were then centrifuged at 13000 g for 5 minutes^14^. One-dimensional^1^H spectra were acquired at 300 K using a standard spin-echo pulse sequence with water suppression and excitation sculpting on a Bruker DRX 500 MHz NMR spectrometer equipped with a cryoprobe. Data were calibrated with respect to the trimethylsilyl propionate (TMSP) signal. Spectra were read into Metabolab^15^ software in Matlab (R2011a, The Math Works, Natick MA) and segmented into 0.005 ppm chemical shift ‘bins’, and spectral areas within each bin were integrated. When used in this paper ppm refers to the spectral position of a given peak, not the quantity of that metabolite. Spectra were corrected for baseline offset and normalised to a total spectral area of unity and a generalised log transformation was applied (26). Binned data were compiled into a matrix and values were normalised using Probabilistic Quotient Normalization (PQN) to account for volume effects^16^. A similar approach, using two-dimensional NMR spectroscopy, was used to compare myoinositol and citrate levels in patients with active renal AAV, urine infection and healthy controls. Quantitative ^1^H-^13^C HSQC (heteronuclar single quantum correlation) spectra were acquired on an Agilent Technologies 18.8 T (800 MHz) DD2 Premium Compact spectrometer with a triple resonance 5 mm enhanced cold probe. Spectra were collected at 25 °C with 12 scans, an initial delay of 3.0 s, a 90° pulse width of 12.1 μs, and an acquisition time of 0.3 s with broadband decoupling.

### Quantification of human urinary metabolites by LC-MS

Human urine samples were obtained from the RKD Biobank (Supplemental Table S3) and comprised patients with small vessel vasculitis (N = 143), healthy controls (N = 45), and non-vasculitic kidney disease controls (N = 23, comprising diabetic nephropathy (n = 5), nephrotic syndrome (n = 5), ischemic nephropathy (n = 5), renal tubular acidosis (n = 2), interstitial nephritis (n = 1) and inflammatory extra-renal disease (endovascular stent infection, diverticulitis, ulcerative colitis, SLE, Henoch-Schonlein purpura). Patients with vasculitis were categorised into remission and active disease using the Birmingham Vasculitis Activity Score (BVAS), with a BVAS score of > 1 indicating active disease, and a positive score in one or more renal BVAS items indicating active renal disease. The UHPLC system consisted of Agilent 1290 Infinity Binary Pump, Sampler, Thermostat and Thermostatted Column Compartment (Agilent Technologies, Waldbronn, Germany). Analytes were retained using an Xbridge Amide column (50 × 2.1 mm i.d., 3.5 μm average total particle diameter; Waters, Cheshire, UK). The column oven temperature was maintained at 40 °C, the autosampler tray at 10 °C and injection volume was 1 μL. Mobile phase flow rate was 0.450 mL/min. Elution programme: 0–1 min 5% A (95% B); 1–7 min 25% A (75% B); 7–7.5 min 50% A (50% B); 7.5–10 min70% A (30% B); 10–15 min 5% A (95% B). Eluent A: aqueous 50 mmol/L ammonium acetate (pH 9.0); eluent B: acetonitrile.

The mass spectrometer used was an Agilent 6495 Triple Quad LC-MS (Agilent Technologies, Santa Clara, USA) equipped with an Agilent Jet Stream electrospray ionisation source, operated in positive ionisation mode (capillary voltage, 2500 V; nozzle voltage, 0 V; capillary gas temperature, 200 °C; capillary gas flow, 15 L/min; sheath gas temperature, 400 °C, sheath gas flow, 11 L/min; nebulizer, 40 psi). The collision energies are shown in Supplemental Table S5. Nitrogen was used as the collision gas. The data was processed using Agilent MassHunter Qualitative analysis software (version B 07.00 SP1), Agilent MassHunter Profinder (version B 06.00) and Mass Profiler Professional (version 12.6.1).

### Statistical analysis of urinary metabolites

Rat metabolomic datasets were statistically analysed using partial-least-squares supervised multivariate methods^17^. All analysis was performed on binned, mean centred spectra using PLS Toolbox (version 5.7) (Eigenvector Research) in Matlab (release 2011a) (Mathworks). PLS-DA was cross-validated using Venetian blinds^18^. PLS weights of individual spectra ppm peaks (reported as absolute values) were used to rank the importance of individual bins.

Partial least squares regression (PLS-R) was used to relate metabolic change to glomerular damage score. The PLS model was tested to determine the optimal number of latent variables (LVs), thereby maximising signal:noise ratio. Metabolites in the resulting LVs were permutation tested to determine the metabolites that were truly predictive with the resulting optimal metabolites forward selected to produce a final model. The resulting model r^2^ value indicates a measure of goodness-of-fit for regression and ranked bins by contribution. Bins were then identified by manual peak fitting of original spectra using Chenomx NMR suite (Chenomx, Alberta, Canada) and MATLAB scripting.

Analysis of human data was performed using SPSS Statistics (version 21, IBM) using a two-step analysis. Metabolite concentrations were scaled to urinary creatinine to reflect typical clinical practice. Metabolite predictors identified by the PLS-DA model with a VIP score > 0.25 were selected and taken forward for binary logistic modelling. The resulting binary logistic model was confirmed using bootstrapping to evaluate predictive power.

## Additional Information

**How to cite this article**: Al-Ani, B. *et al*. Changes in urinary metabolomic profile during relapsing renal vasculitis. *Sci. Rep.* **6**, 38074; doi: 10.1038/srep38074 (2016).

**Publisher's note:** Springer Nature remains neutral with regard to jurisdictional claims in published maps and institutional affiliations.

## Supplementary Material

Supplementary Information

## Figures and Tables

**Table 1:** Urinary metabolite profile of rats with EAV at day 56 and day 210.

ppm	Metabolite	D56		D210	
		PLS-DA weight	PLS-R score	PLS-DA weight	PLS-R score
3.2680, 3.2739	TMAO	**▴0.63**	**11.68**	**▴**0.17	**12.92**
2.4466, 3.0157 2.4349, 2.4408	2-Oxoglutarate	**▴0.23**	**11.21**	**▴0.37**	3.50
2.6989, 2.5757 2.5522	Citrate	▾0.17	5.43	▾**0.39**	3.58
2.9277	Dimethylglycine	**▴**0.14	3.15		
2.4114	Succinate	▾0.13	**10.44**		
3.8547	Homoserine	**▴**0.13			
3.2621	Betaine	**▴**0.11			3.03
3.4323, 3.7021, 3.8253	Maltose/Glucose		5.07		
3.8312	Maltose				3.65
2.5464	Beta-alanine			**▴**0.15	
2.6930	Carnosine			**▴**0.14	
2.7282, 2.7048	Dimethylamine			**▴**0.13	**3.92**
3.6082, 3.2503	myo-Inositol	**▴**0.10	1.64		2.25

Top-ranked metabolites are shown for analysis at each time point. All comparisons are control versus test; therefore ▴ indicates increase in MPO immunised (v HSA immunised) rats at day 56, and MPO relapsed (v saline treated, MPO-immunised animals) at day 210. The top contributing metabolites in each analysis are highlighted. Shown values are weights for PLS-DA and scores for PLS-R.

**Table 2:** Binary logistic regression of ANOVA selected metabolites and existing markers at day 56 in rat urine.

Predictors	B	S.E.	Wald	df	Sig.	Exp (B)	Model Accuracy			
							HSA	MPO	All	p
Haematuria	1.771	0.963	3.381	1	0.066	5.875	75	100	90.5	<0.001
ACR	0.014	0.03	0.217	1	0.642	1.014				
Haematuria	2.212	1.251	3.127	1	0.077	9.133	87.5	100	95.2	0.001
ACR	−0.004	0.063	0.005	1	0.946	0.996				
DMG	2.029	18.514	0.012	1	0.913	7.604				
TMAO	4.311	5.6	0.593	1	0.441	74.507				

A predictive model built with existing markers (top panel) is strongly predictive (90.5% accuracy p < 0.001) of HSA and MPO immunisation status. Adding metabolites (lower panel) raises prediction capacity by 5%.

**Table 3:** Binary logistic regression of ANOVA selected metabolite and existing markers in MPO immunised animals at day 210.

Predictors	B	S.E.	Wald	df	p	OR	Model Accuracy			
							MPO	Control	Total	p
Haematuria	0.46	0.909	0.256	1	0.613	1.584	20	85.7	58.3	0.665
ACR	−0.004	0.005	0.718	1	0.397	0.996				
Haematuria	0.171	1.168	0.021	1	0.884	1.187	80	85.7	83.3	0.122
ACR	−0.003	0.01	0.074	1	0.785	0.997				
2-OG	−13.29	8.447	2.475	1	0.116	0				
2-OG	−13.36	7.432	3.231	1	0.072	0	80	85.7	83.3	0.017

A predictive model built with existing markers is poorly predictive (58.3% accuracy p = 0.665) of relapse status in EAV (top panel). Adding 2-oxoglutarate (2-OG) to the model improved prediction and p values slightly but not to significance (middle panel). Removal of the current biomarkers, leaving 2-oxoglutarate alone resulted in a statistically significant model predicting 83.3% of relapse status (lower panel). OR = Odds ratio.

**Table 4:** ANOVA of all creatinine-normalised metabolites analysed in human urine by LC-MS, ranked by their association with active renal vasculitis.

	Sum of Squares	Mean Square	F	Sig.
myo-Inositol	0	0	55.879	0
Citric acid	0.044	0.044	12.379	0.001
Maltose	0	0	8.357	0.005
Succinate	0	0	5.603	0.019
2-oxoglutarate	0	0	1.705	0.194
Betaine	0.002	0.002	1.124	0.291
TMAO	0.001	0.001	0.193	0.661
Glutaric acid	0	0	0.187	0.666
Dimethylglycine	0	0	0.031	0.861

Summary statistics shown are for between-groups comparisons.

**Table 5:** Binary logistic models using existing biomarkers and novel metabolites in human urine to predict active renal vasculitis.

Existing measure	B	S.E.	Wald	Sig.	Std OR	95% CI								
CRP	0.011	0.007	2.746	0.004	2.288	1.31	4.00							
eGFR	−0.059	0.03	3.89	<0.001	0.367	0.21	0.63							
PCR	0.017	0.007	5.349	0.007	3.238	1.38	7.62							
*Model constant*	*−0.486*	*1.457*	*0.111*	*0.739*	*0.156*									
**Metabolite**	**B**	**S.E**	**Wald**	**Sig.**	**Std OR**	**95% CI**								
*Citric acid*	−39.65	11.8	11.186	0.001	0.067	0.014	0.33							
myo-inositol	6039	1536.5	15.452	<0.001	9.245	3.05	28.0							
*Model constant*	*−0.046*	*0.675*	*0.005*	*0.945*	*0.955*									

Existing biomarkers (CRP, eGFR, PCR) were used for the base model. Identified metabolite predictors were selected by forward selection from the identified candidates (myo-Inositol, citric acid, glycolic acid, maltose and succinate) based on their ability to significantly improve the model. Std OR = Standardised odds ratio.
