# Supplementary material for: Changes in urinary metabolomic profile during relapsing renal vasculitis
Source: Sci Rep. 2016 Dec 1;6:38074. doi: 10.1038/srep38074 (PMC5131479; doi:10.1038/srep38074)
Supplement: Supplementary Information [file srep38074-s1.pdf]

Changes in urinary metabolomic profile during relapsing renal vasculitis

Bahjat Al Ani[1,3], Martin Fitzpatrick[2], Hamad Al Nuaimi[1], Alice M Coughlan[4], Fionnuala B Hickey[4], Charles D Pusey[5], Caroline Savage[1], Christopher M Benton[6], Eóin C O'Brien[4], Declan O'Toole[7], K. H. Mok[7], Stephen P Young[2], Mark A Little[8*]

[1]Renal Immunobiology Group, School of Infection, Immunology and Inflammation, University of Birmingham, UK

[2]Rheumatology Research Group, Centre for Translational Inflammation Research, College of Medical and Dental Sciences, University of Birmingham, UK

[3]Current address: Department of Physiology, College of Medicine, King Khalid University, Abha 62529, Saudi Arabia.

[4]Department of Clinical Medicine, Trinity College Dublin, Ireland

[5]Renal Section, Imperial College London, London, UK

[6]Agilent Technologies Ltd, UK.

[7]School of Biochemistry and Immunology, Trinity Biomedical Sciences Institute (TBSI), Trinity College Dublin, Ireland

[8]Trinity Health Kidney Centre, Trinity Centre for Health Sciences, Tallaght Hospital, Dublin 24. Ireland

***Address correspondence to:**

Prof Mark Little

Trinity Health Kidney Centre, Trinity Centre for Health Sciences, Tallaght Hospital, Dublin 24, Ireland.

Tel: +353-1-896-2145 Email: mlittle@tcd.ie

Supplemental Figures

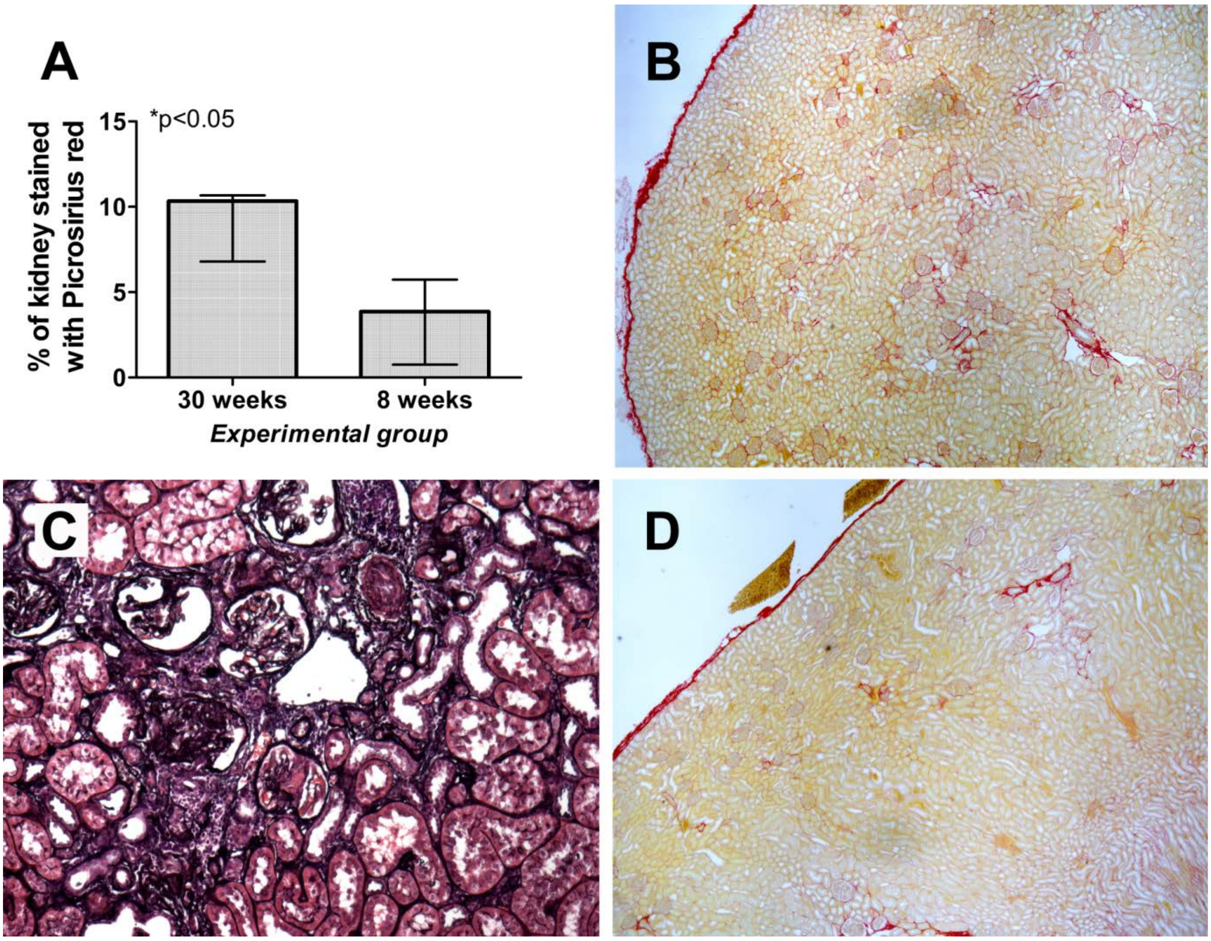

Supplemental Figure S1. **Renal scarring is evident at day 210 (week 30) after induction of EAV**. WKY rats were immunised with hMPO or HSA and sacrificed at day 56 (week 8) or day 210 (week 30). The degree of renal scarring was assessed by staining tissue with picrosirius red and PAMS. **(A)** Picrosirius red staining was quantified by blinded image analysis. The median fraction of kidney tissue staining red was higher in animals analysed at day 210 than at the peak of acute glomerulonephritis at day 56. Data are presented as the median and IQR. **(B, D)** Representative images of picrosirius red stained kidney after **(B)** 210 days and **(D)** 8 weeks (x4). **(C)** Representative kidney section stained with PAMS in a rat with EAV sacrificed at day 210 (x20). Fibrous tissue is stained black.

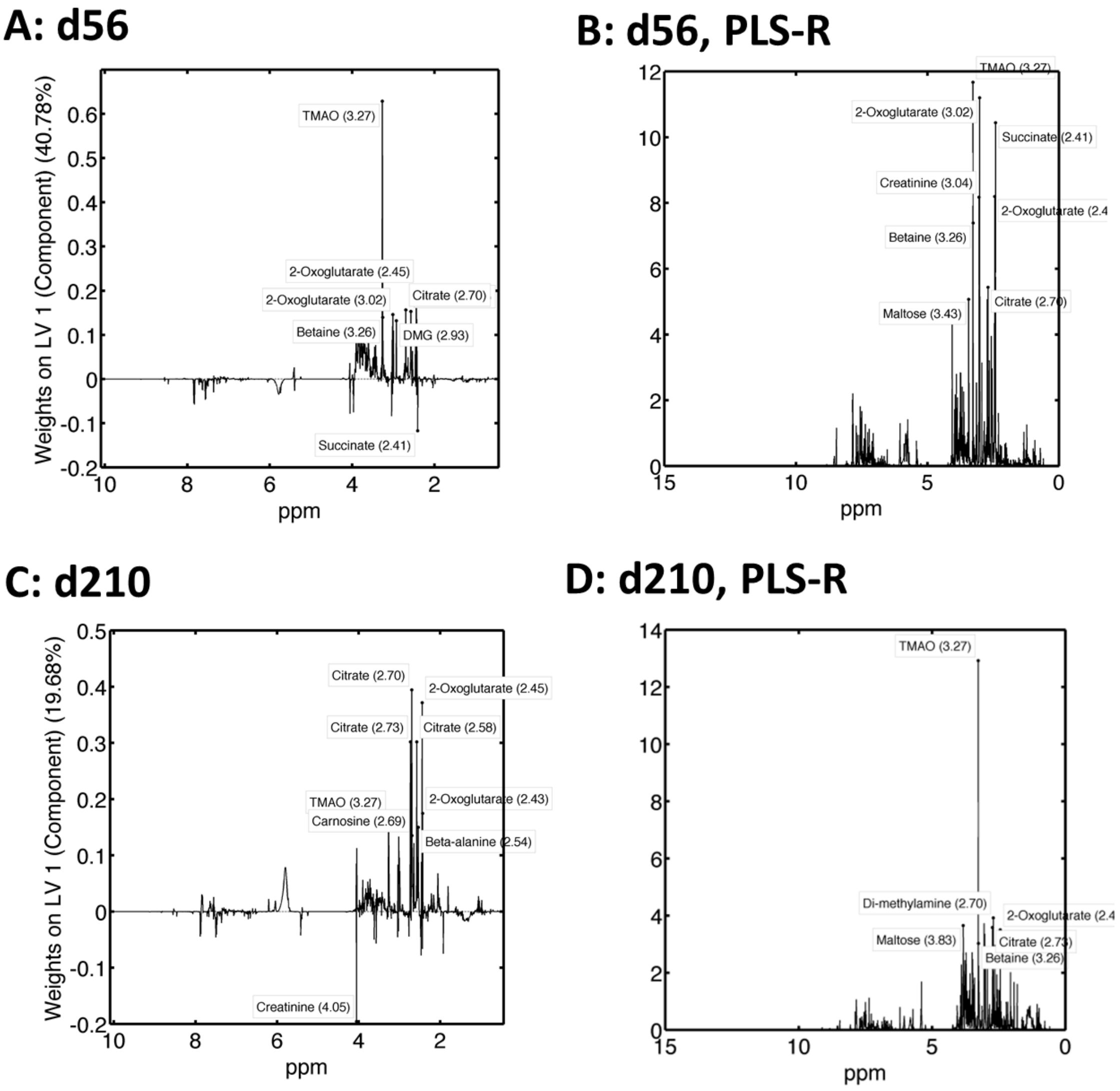

Supplemental Figure S2. Binned 1D NMR spectra of rat urine at point of peak disease (day 56) and following induction of relapse with MPO/LPS (day 210). (**A**) PLS-DA weights plot for LV 1 showing key contributing NMR peaks to separation between HSA and MPO treated rats at day 56, with positive values indicating metabolites raised in MPO treated animals. Treated rats had increased TMAO, 2-oxoglutarate, citrate, betaine and DMG. (**B**) PLS-R rank plot of urinary NMR peaks at day 56 found to be positively or negatively correlated with histological glomerular damage score. Predictive peaks are labelled, including TMAO, 2-oxoglutarate, succinate, betaine, citrate and maltose. (**C**) PLS-DA weights plot for LV 1 showing key contributing NMR peaks to separation between MPO re-stimulated and saline treated rats at day 210, with positive values indicating metabolites raised in

MPO treated animals. Treated rats had increased citrate, 2-oxoglutarate, TMAO, carnosine and beta-alanine. (**D**) PLS-R rank plot of urinary NMR peaks at day 210 found to be positively or negatively correlated with histological glomerular damage score. Predictive peaks are labelled, including TMAO, dimethylamine, 2-oxoglutarate, maltose, citrate and betaine.

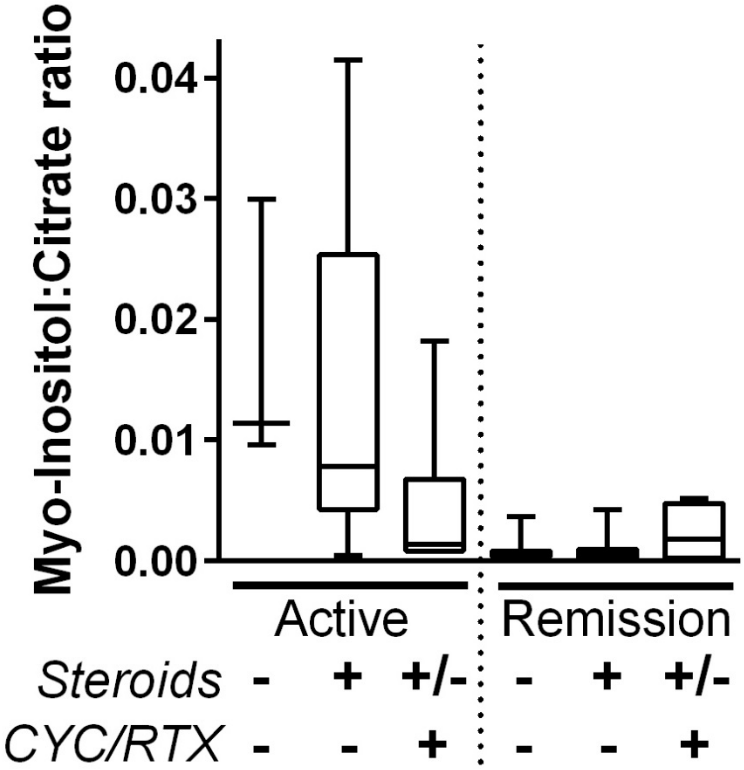

Supplemental Figure S3. Comparison of the effect of treatment on urine myo-inositol:citrate ratio in the urine of patients with active renal vasculitis and those in remission. CYC=Cyclophosphamide; RTX=Rituximab; +/- implies that the group contains patients both receiving and not receiving corticosteroid therapy.

Supplemental Tables

Predictor	Sum of Squares	df	F	Sig.
**Haematuria**	22.77	1	35.511	0
**DMG**	0.10	1	5.915	0.025
**TMAO**	1.89	1	5.651	0.028
**ACR**	48389	1	4.78	0.042
**2-oxoglutarate**	0.31	1	2.808	0.11
**Citrate**	0.53	1	1.592	0.222
**Succinate**	0.01	1	0.661	0.426

Supplemental Table S1. ANOVA of key day 56 factors discriminating between MPO and HSA immunised rats. The existing markers (haematuria and ACR) are both significant ($p<0.001$ and $p<0.05$ respectively). Novel metabolite biomarkers DMG and TMAO are also significant discriminators at this time point ($p<0.05$).

Predictor	Sum of Squares	df	F	Sig.
**2-oxoglutarate**	0.09	1	7.11	0.024
**Citrate**	0.12	1	1.693	0.222
**TMAO**	0.04	1	0.502	0.495
**ACR**	13348.41	1	0.475	0.506
**Succinate**	0.002	1	0.255	0.625
**Haematuria**	0.01	1	0.012	0.913
**DMG**	0	1	0	0.989

Supplemental Table S2. ANOVA of key day 210 factors discriminating between animals relapsed with MPO and vehicle. The existing markers (haematuria and ACR) were poor predictors at this time point (both $p>0.05$). Of the putative urine metabolite biomarkers only 2-oxoglutarate was a significant predictor in this analysis.

	Vasculitis patients	Disease Controls	Healthy Controls	p-value
n	143	23	45	
Age (median, range)	63.3 (21-90)	62.2 (16-87)	53.9 (20-76)	0.002
Male, n (%)	76 (53.1)	11 (47.8)	15 (30.0)	0.001
Diagnosis, n (%)				
GPA	82 (57.3)	NA	NA	
MPA	44 (30.8)	NA	NA	
EGPA	10 (6.9)	NA	NA	
Anti-GBM disease	3 (2.0)	NA	NA	
Double Positive[#]	4 (2.8)	NA	NA	
ANCA specificity, n (%)				
Proteinase-3	81 (60)	NA	NA	
Myeloperoxidase	54 (40)	NA	NA	
Disease Characteristics, n (%)				
Active (renal)	28 (19.6)	NA	NA	
Active (extra-renal)	11 (7.7)	NA	NA	
Remission	104 (72.8)	NA	NA	
Kidney function, n (%)				0.5*
eGFR <30	25 (17.5)	3 (13.0)		
eGFR 30-60	44 (30.8)	10 (43.5)		
eGFR >60	74 (51.8)	10 (43.5)	45 (100)	
Dialysis	10 (6.9)	0 (0)	0 (0)	
Immunosuppressive treatment, n%				
Corticosteroids	88 (61.5)	4 (17.4)	0	
Cyclophosphamide	10 (7.0)	0 (0)	0	
Rituximab	6 (4.2)	0 (0)	0	
Other	56 (39.2)~	4 (17.4)	0	
None	42 (29.4)	17 (73.9)	0	

Supplemental Table S3. Details of cases used in human LC-MS analysis. *Comparing disease controls and vasculitis cases. [#]Double positive for both ANCA and anti-GBM. ~Azathioprine, methotrexate or mycophenolate mofetil, NA = Not applicable

	B	Std OR	Bias	S.E.	Sig	95 C.I.	
**Citric acid**	-39.65	0.07	-9.704	32.382	0.037	-9.21	-0.87
**Myo-Inositol**	6039	9.24	1263.52	3322.589	0.001	1.44	5.72
**Model constant**	-0.046	0.05	0.184	1.157	0.973	-8.92	-1.72

Supplemental table S4. Binary logistic model of metabolite predictors with bootstrapping. The established model was bootstrapped (997 samples) to improve estimates of model accuracy. Bootstrapping confirmed predictors that were significantly altered in active renal vasculitis compared to cases in remission.

Compound	Precursor ion (m/z)	Product ion (*m/z*)	Dwell Time (ms)	Collision Energy (V)	Cell Accelerator (V)	Polarity
**N-Phenylacetylglycine**	194.1	91.05	20	15	2	+
**N-Phenylacetylglycine**	194.1	76.04	20	15	2	+
**Betaine**	118.09	59.07	20	25	2	+
**Betaine**	118.09	58.06	20	25	2	+
**Creatinine**	114.1	86.1	20	10	2	+
**Creatinine**	114.1	72	20	10	2	+
**Creatinine**	114.1	44.1	20	40	2	+
**Dimethylglycine**	104.07	58.06	20	15	2	+
**Dimethylglycine**	104.07	42.03	20	15	2	+
**TMAO**	76.1	58.1	20	20	2	+
**Maltose**	341.1	221.07	20	15	2	-
**Maltose**	341.1	161.01	20	5	2	-
**Maltose**	341.1	101.02	20	15	2	-
**Citric Acid**	191.02	111	20	10	2	-
**Citric Acid**	191.02	87	20	15	2	-
**Myo Inositol**	179.06	161	20	10	2	-
**Myo Inositol**	179.06	87	20	15	2	-
**Oxoglutaric Acid**	145	101	20	10	2	-
**Oxoglutaric Acid**	145	73	20	10	2	-
**Oxoglutaric Acid**	145	57	20	10	2	-
**Glutaric Acid**	131.03	113	20	10	2	-
**Glutaric Acid**	131.03	87	20	10	2	-
**Succinate**	117.02	99	20	10	2	-
**Succinate**	117.02	73	20	10	2	-
**Glycolic Acid**	75	75	20	0	2	-
**Glycolic Acid**	75	57	20	3	2	-

Supplemental Table S5. Collision energies used in LC-MS analysis.
